# Molecular and Physiological Effects on the Small Intestine of Weaner Pigs Following Feeding with Deoxynivalenol-Contaminated Feed

**DOI:** 10.3390/toxins10010040

**Published:** 2018-01-12

**Authors:** J. Alex Pasternak, Vaishnavi Iyer Aka Aiyer, Glenn Hamonic, A. Denise Beaulieu, Daniel A. Columbus, Heather L. Wilson

**Affiliations:** 1 Vaccine and Infectious Disease Organization-International Vaccine Centre (VIDO-InterVac), University of Saskatchewan, Saskatoon, SK S7N 5E3, Canada; alex.pasternak@usask.ca (J.A.P.); glenn.hamonic@usask.ca (G.H.); 2 Prairie Swine Centre, Inc., Saskatoon, SK S7N 5E3, Canada; vaishiyer95@gmail.com (V.I.A.A.); dan.columbus@usask.ca (D.A.C.); 3 Department of Animal and Poultry Science, University of Saskatchewan, Saskatoon, SK S7N 5E3, Canada; denise.beaulieu@usask.ca

**Keywords:** ileum, jejunum, deoxynivalenol, piglet, contaminated feed, tight junction, Relative to control piglets receiving 0.20–0.40 ppm deoxynivalenol (DON)-contaminated feed, weaner piglets fed 3.30–3.80 ppm DON for 24 d had significantly reduced body weight. However we observed no significant impact on average daily feed intake, average daily gain, serum or gut cytokine expression (with exception of elevated ileal Claudin-7), gut morpholoyg, or histology.

## Abstract

We intended to assess how exposure of piglets to deoxynivalenol (DON)-contaminated feed impacted their growth, immune response and gut development. Piglets were fed traditional Phase I, Phase II and Phase III diets with the control group receiving 0.20–0.40 ppm DON (referred to as the Control group) and treatment group receiving much higher level of DON-contaminated wheat (3.30–3.80 ppm; referred to as DON-contaminated group). Feeding a DON-contaminated diet had no impact on average daily feed intake (ADFI) (*p* < 0.08) or average daily gain (ADG) (*p* > 0.10) but it did significantly reduce body weight over time relative to the control piglets (*p* < 0.05). Cytokine analysis after initial exposure to the DON-contaminated feed did not result in significant differences in serum interleukin (IL) IL1β, IL-8, IL-13, tumor necrosis factor (TNF)-α or interferon (IFN)-γ. After day 24, no obvious changes in jejunum or ileum gut morphology, histology or changes in gene expression for IL-1β, IL-6, IL-10, TNFα, or Toll-like receptor (TLR)-4 genes. IL-8 showed a trend towards increased expression in the ileum in DON-fed piglets. A significant increase in gene expression for claudin (CLDN) 7 gene expression and a trend towards increased CLDN 2-expression was observed in the ileum in piglets fed the highly DON-contaminated wheat. Because CLDN localization was not negatively affected, we believe that it is unlikely that gut permeability was affected. Exposure to DON-contaminated feed did not significantly impact weaner piglet performance or gut physiology.

## 1. Introduction

Deoxynivalenol (DON), commonly known as vomitoxin, is a potent mycotoxin produced by the fungus *Fusarium graminearum*, and its presence in wheat, corn, and barley crops can lead to them being downgraded to livestock feed grade. Pigs, and in particular young piglets, are poorly tolerant to DON contamination. Although extremely high doses of contamination in feed (20 mg/kg feed) will induce vomiting [1,2], swine will tolerate lower-level feed contamination to varying degrees in a sex- and dose-dependent manner [3]. Longer-term exposure to moderate contamination of feed at levels between 5 and 8 mg/kg will be tolerated but has been shown to considerably decrease daily feed intake and growth rate [4,5]. As a result, governmental guidelines from the Canadian Food Inspection Agency, United States Food and Drug Administration and the European Union recommend limiting dietary inclusion in swine feed to under 1 mg/kg [6], 1 mg/kg [7], and 0.9 mg/kg [8] respectively. However even at these recommended inclusion levels DON has been shown to significantly decrease average daily gain (ADG) and alter intestinal morphology [9]. The majority of research to date has focused on the local effect of DON on the intestine but doses, age of animal, and exposure times have varied which makes it difficult to compare results. In vivo studies have demonstrated that chronic exposure to 3 mg/kg DON-contaminated feed altered intestinal morphology including villus atrophy and reduced villi height, reduced jejunal and ileal goblet cells and lymphocytes counts, as well as reduced expression of junctional adheren protein E-cadherin and tight-junction protein occludin in the intestine [10]. Several studies show that piglets fed DON had altered cytokine production either in the duodenum, jejunum, or the ileum or the mesenteric lymph nodes [9,11,12] indicating that DON-contaminated feed can alter the innate immune response in a piglet’s gut. With increased quantities of DON-contaminated grain entering the livestock sector, complete avoidance of DON may not be possible.

The intestinal tract is the first physical barrier to protect the body from food contaminants, chemicals and intestinal pathogens. A single layer of epithelial cells separates the apical and basolateral domains of the gut mucosa. Tight junctions (TJs) between adjacent cells are regulated by structural and functional proteins including Occluden, Junction Adhesion molecules and Claudin family members, which together regulate permeability through the intercellular space on epithelial sheets [13,14,15,16,17]. How DON reportedly affects barrier function and specifically the proteins involved in TJ formation is variable based on experimental design, age of animals, amount of DON present and duration of exposure [10,18]. It is, therefore, neccessary to better understand the physiological effects underlying the reduced performance by pigs consuming DON-contaminated diets in order to develop effective and economical strategies.

We sought to clarify how weaner piglets fed traditional Phase I, Phase II and Phase III diets with the control group receiving 0.30 ppm DON and treatment group receiving 3.30 ppm DON in Phase I, control group receiving 0.20 ppm DON and treatment group receiving 3.80 ppm DON in Phase II, and control group receiving 0.40 ppm DON and treatment group receiving 3.80 ppm DON in Phase III were affected. We measured ADG, average daily feed intake (ADFI), and gene expression profiles for innate immune response receptors and cytokines, as well as genes that play a role in intestinal barrier function. Immunohistofluorescence was performed to establish localization of several proteins that mediate TJ formation in the jejunum and the ileum. This research will help to establish whether homeostatic mechanisms compensate for DON exposure in vivo over the long term.

## 2. Results

### 2.1. Feed Intake and Growth Performance

No pigs showed any signs of vomiting throughout the trials. One pig from the control diet died during the study due to reasons unrelated to dietary treatments. Body weight (Table 1) was not different between groups (*p* > 0.05) up to day 24 of the study but final body weight was significantly reduced (*p* < 0.05) in pigs fed the DON diet.

Average daily gain and average daily feed intake were not affected (Table 2, *p* > 0.05) in the first three weeks of the study period. There was a trend (*p* < 0.08) for average daily feed intake to be reduced in pigs fed the DON diet in the final days of the study.

### 2.2. Serum Cytokine Analysis in Acute Period after DON Exposure

**Figure 1:**
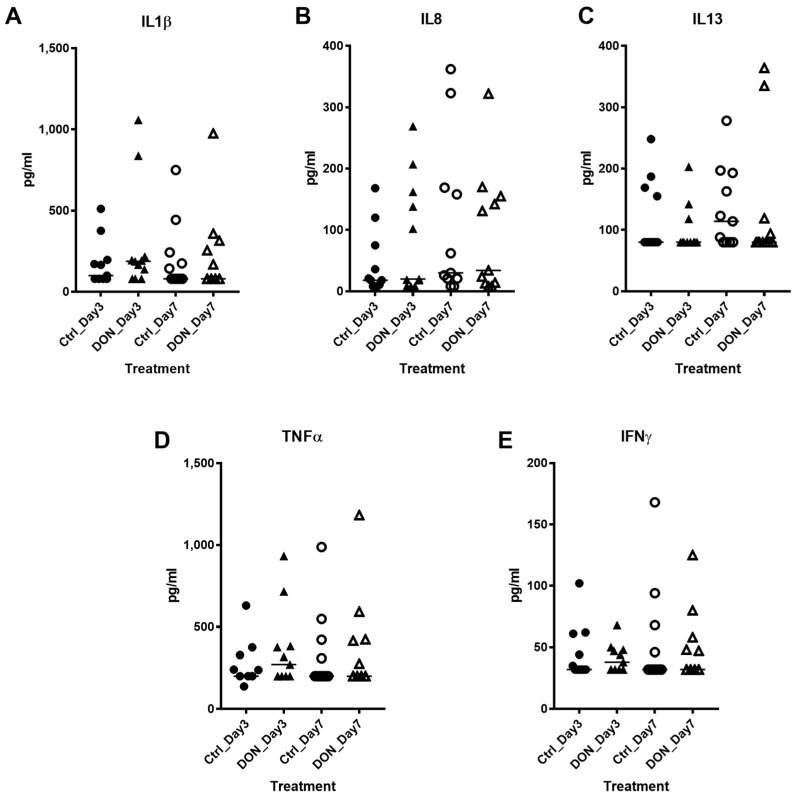
Acute but low-level exposure of DON-contaminated feed did not impact serum cytokine production. Sera were collected on Day 3 and Day 7 and subjected to BioPlex analysis to assess changes in (**A**) IL-1β, (**B**) IL-8, (**C**) IL-13, (**D**) TNF-α and (**E**) IFN-γ in response piglets fed DON-contaminated feed or Control feed after 3 and 7 days. Each data point represents a unique biological replicate and the horizontal line represents the median value for the group. Statistical analysis was performed with a nonparametric Kruskal-Wallis between DON and Control fed piglets on Day 3 and Day 7 as well as within each treatment group over time.

Three and seven days after introduction of the Phase I diets, sera were collected and systemic cytokines levels were assessed (Figure 1). We did not identify a significant difference in serum IL-1β (Figure 1A), IL-8 (Figure 1B) IL-13 (Figure 1C), TNF-α (Figure 1D) or IFN-γ (Figure 1E) between animals fed the Control or DON-contaminated diets after 3 or 7 d. We also did not detect a significant change in any of the serum cytokines over time within each treatment group. These data indicate that under the current experimental conditions, DON-contaminated feed did not promote a systemic inflammatory immune response.

### 2.3. Jejunal and Ileal Immune Response Gene Profile after Exposure to DON

**Figure 2:**
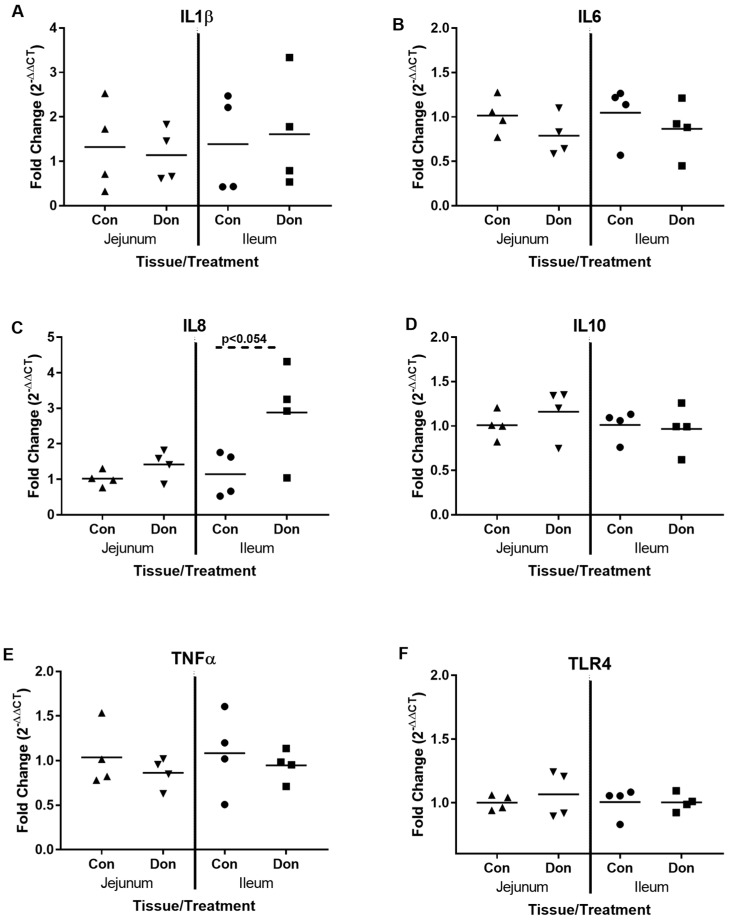
QPCR analysis of cytokines and TLR4 in jejunal and ileal gut tissue. After 24 days of exposure, jejunal and ileal gut samples from control and DON-fed piglets were investigated for relative expression of IL-1β (**A**), IL-6 (**B**), IL-8 (**C**), IL-10 (**D**), TNFα (**E**) and TLR4 (**F**) mRNA expression. The mRNA expression levels of each gene were normalized with the housekeeping genes and were calculated with 2^−ΔΔCt^ relative quantification. Each data point represents a unique biological replicate. Horizontal bars represent the median values.

Next, we wanted to assess how DON exposure for 24 days affected piglet cytokine gene expression in the jejunum and the ileum. In the jejunum, we observed no significant difference in the expression of IL-1β (Figure 2A), IL-6 (Figure 2B), IL-8 (Figure 2C), IL-10 (Figure 2D), and TNFα (Figure 2E) genes in control or DON-fed piglets. These same cytokines also showed no change in expression in the ileal tissue between the Control and DON-fed piglets (*p* > 0.10), with the exception of IL-8, which showed a trend towards increased expression in the DON-treated tissues (Figure 2C; *p* < 0.06). Likewise, the gene for TLR4 that codes for a receptor that detects lipopolysaccharide from Gram negative bacteria was not differentially expressed between the Control and DON-fed piglets (Figure 2F).

**Figure 3:**
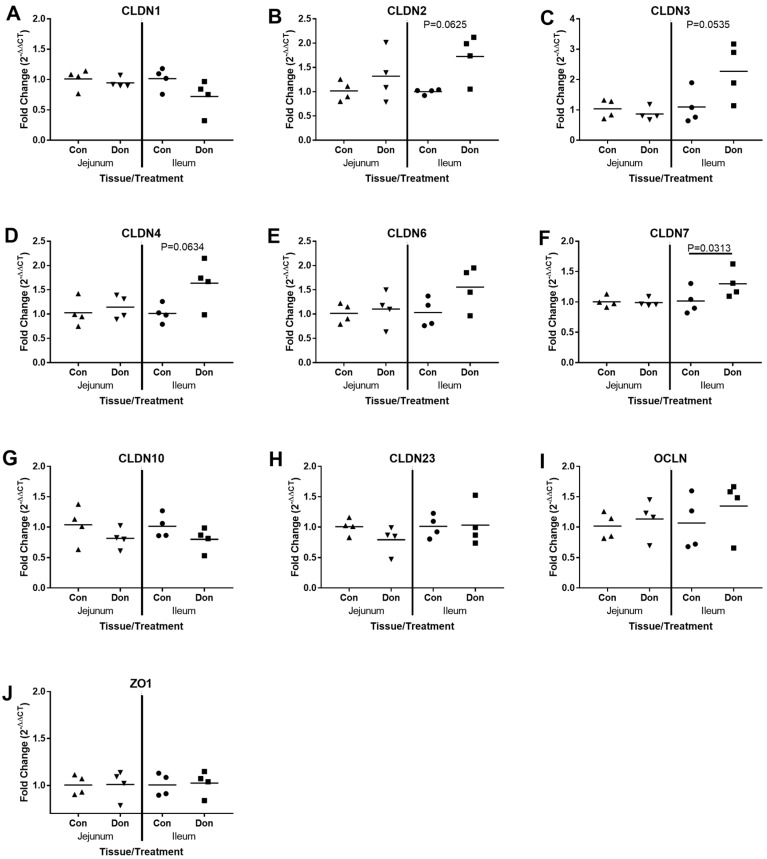
QPCR analysis of Claudins, Occluden and Zonodulin 1 in jejunal and ileal gut tissue. After 24 days of exposure, jejunal and ileal gut samples from control and DON-fed piglets were investigated for relative expression of Claudin (CLDN)-1 (**A**), (CLDN)-2 (**B**), (CLDN)-3 (**C**), (CLDN)-4 (**D**), (CLDN)-6 (**E**), (CLDN)-7 (**F**), (CLDN)-10 (**G**), (CLDN)-23 (**H**), Occluden (OCLN) (**I**), and Zonodulin-1 (ZO1) (**J**) mRNA expression. The mRNA expression levels of each gene were normalized with the housekeeping genes and were calculated with 2^−ΔΔCt^ relative quantification. Each data point represents a unique biological replicate. Horizontal bars represent the median values.

In recent years, occludens, junctional adhesion molecule proteins, claudin family members and others have been shown to be responsible for mediating TJ formation [19]. We investigated whether DON exposure could influence the expression profile of several genes, which encode TJ proteins including Claudins (CLDNs), Occluden (OCLN) and Zonula occludens-1 (ZO-1) in jejunal and ileal tissue. CLDN-1 expression (Figure 3A) was not significantly altered in response to DON but CLDN2 (Figure 3B; *p* < 0.063), CLDN-3 (Figure 3C; *p* < 0.054), and CLDN-4 (Figure 3D; *p* < 0.063) showed a trend towards upregulation in the ileum (but not the jejunum) in the DON-treated animals relative to age-matched Control-fed piglets. Expression of CLDN-7 was significantly induced in the ileal tissue from DON-treated animals (Figure 3F; *p* < 0.031) but no change in expression was observed in the jejunum relative to the Control-fed piglets. We observed no significant difference in gene expression for CLDN-10 (Figure 3G), CLDN-23 (Figure 3H), OCLN (Figure 3I) or ZO-1 (Figure 3J) in either tissue across treatment groups. Gene expression analysis for CLDN-8 and CLDN-14 were assessed however expression of these transcripts was below the threshold of detection (data not shown). DON-contaminated feed had no effect on expression patterns of the indicated genes in the jejunum and only modest effect on expression in the ileum.

**Figure 4:**
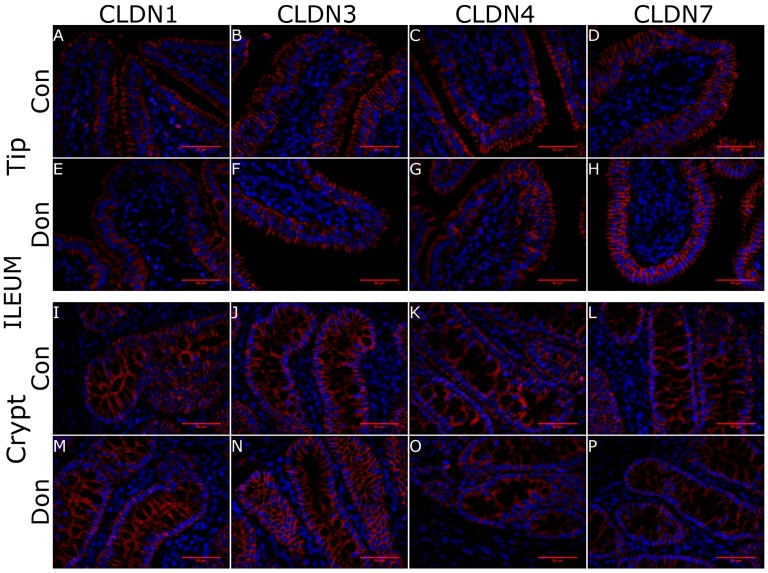
Claudin surface localization in piglet ileal villi and crypts in DON-fed and control fed piglets. Ileal tissue was obtained 24 days after DON-exposure to half of the piglets. CLDN1 was localized to the full length of the pericellular junction within the crypts where as it was found more heavily localized to the apical aspect of the pericellular junction at the villus tip (**A**,**E**,**I**,**M**). CLDN3 stained the length of the pericellular junction at the villus tip and within the crypts but was more abundant in the latter (**B**,**F**,**J**,**N**). CLDN4 stained the villous surface but was found intracellularly localized in the epithelium of the crypts (**C**,**G**,**K**,**O**). CLDN7 stained along the length of the pericellular junction at both the villus tip and within the crypts (**D**,**H**,**L**,**P**). Secondary antibody: Alexa555-conjugated goat α rabbit IgG (red) in incubation buffer for 4 h at room temperature. Nuclear stain: DAPI (blue). Scale bar represents 50 μm.

**Figure 5:**
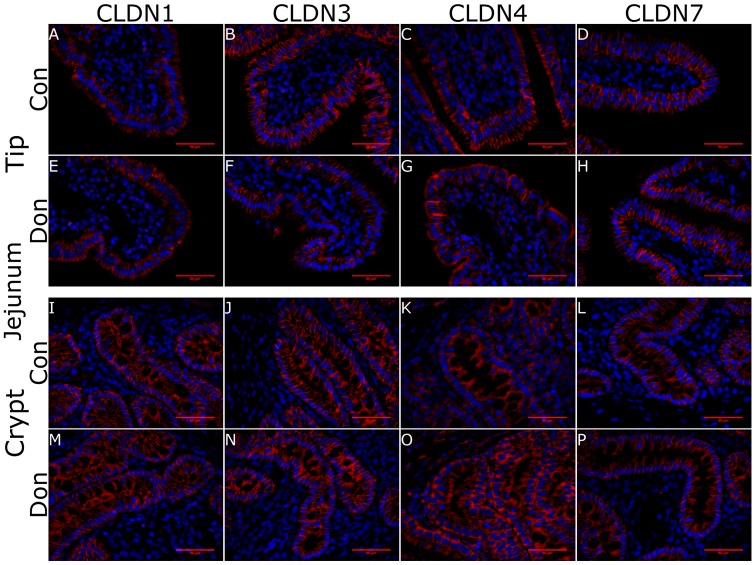
Claudin surface localization in piglet jejunal villi and crypts in DON-fed and control fed piglets. Jejunal tissue was obtained 24 days after DON-exposure to half of the piglets. CLDN1 was localized to the full length of the pericellular junction within the crypts where as it was found more heavily localized to the apical aspect of the pericellular junction at the villus tip (**A**,**E**,**I**,**M**). CLDN3 stained the length of the pericellular junction at the villus tip and within the crypts but was more abundant in the latter (**B**,**F**,**J**,**N**). CLDN4 stained the villous surface but was found intracellularly localized in the epithelium of the crypts (**C**,**G**,**K**,**O**). CLDN7 stained along the length of the pericellular junction at both the villus tip and within the crypts (**D**,**H**,**L**,**P**). Secondary antibody: Alexa555-conjugated goat α rabbit IgG (red) in incubation buffer for 4 h at room temperature. Nuclear stain: DAPI (blue). Scale bar represents 50 μm.

Next, we assessed whether DON-contaminated feed impacted villous or crypt morphology (using H & E staining) or surface localization of CLDN-1, CLDN-3, CLDN-4, CLDN-7 proteins in ileum villi (Figure 4A–H) and crypts (Figure 4I–P) and jejunal villi (Figure 5A–H) and crypts (Figure 5I–P) relative to control fed piglets using immunohistofluorescence. We observed no change in villous or crypt morphology per villi in piglets fed DON-contaminated or control feed (data not shown). In both regions of the gut, CLDN1 was localized to the full length of the pericellular junction within the crypts (Figure 4I,M and Figure 5I,M) where it was found more heavily localized to the apical aspect of the pericellular junction at the villus tip (Figure 4A,E and Figure 5A,E). CLDN1 was also expressed in intestinal endothelial cells (data not shown). CLDN3 stained the length of the pericellular junction at the villus tip (Figure 4B,F and Figure 5B,F) and within the crypts (Figure 4J,N and Figure 5J,N). CLDN4 staining at the villus tip was observed along the length of the pericellular junction (Figure 4C,G and Figure 5C,G) whereas it was found intracellularly localized in the epithelium of the crypt for both control fed and DON-fed piglets (Figure 4K,O and Figure 5K,O). CLDN7 stained along the length of the pericellular junction at both the villus tip (Figure 4D,H and Figure 5D,H) and within the crypts (Figure 4L,M and Figure 5L,M). The figures shown are representative of 4 biological replicates (Supplementary Figures S1–S8). We note that the IHF staining intensity was strongest for CLDN7 >>> CLDN3 > CLDN4 > CLDN1 which is not obvious from the figures because specific imaging protocols were used to evaluate each anti-CLDN antibody (data not shown). IHC analysis of this panel of CLDNs indicates that exposure of piglets to DON-contaminated feed did not negatively impact CLDN localization in jejunal and ileal villi or crypts relative to those fed control feed.

## 3. Discussion

The aim of this study was to determine whether the piglet gut can compensate for DON-contaminated feed by showing gut health and strong growth kinetics. Most studies show that piglets fed DON-contaminated feed have altered gut histology and reduced performance. For instance, jejunal explants from 4 to 5 week old piglets and 9–13 week old pigs exposed to 5 μM DON (corresponds to 1.5 mg/kg in diet) for 8 hours were shown to have shortened intestinal villi and lysed intestinal cells however the younger piglets were shown to have better morphological scores [20]. However, no effect on morphological scores was observed in 4–5 week old piglet gut explants exposed for 4 h to 1 μM DON (which corresponds to 0.3 mg DON/kg in diet) [20]. In vivo studies showed 0.9–2.29 mg/kg DON in feed resulted in shortening of villi and morphological effects [21]. In contrast, our results showed piglets fed up to 3.80 ppm DON-contaminated feed had reduced ADG and a tendency towards reduced ADFI relative to the control pigs that were exposed to up to 0.40 ppm DON, but only in the last days of the trial. The jejunum and ileum showed no significant changes in villous or crypt architecture between our control and DON-fed groups. We speculate that the low level DON contamination in the control diet may have had an impact on the gut, which makes it difficult to observe a difference between this diet and the treatment diet with 3.80 ppm DON.

How DON affects barrier function and specifically the proteins involved in TJ formation is unknown and results have been variable, possibly due to differences in experimental design, age of animals, amount of DON present, and duration of exposure. Using Ussing chambers to investigate jejunal tissues, it was determined that 2–3 month old pigs fed 4–8 mg/mL DON showed inhibited active transport of nutrient across the small intestinal wall [22]. Others [10] showed that 5-week old piglets fed 3 mg DON /kg feed for 35 days did not show significantly reduced weight but there was reduced adherent junction protein E-cadherin and the tight junction protein occludin in the intestine. Similarly, 4-week old piglets fed 0.9 mg DON/kg feed for 10 days showed reduced mRNA expression of occludin in the intestine [9]. Immunohistochemistry of the jejunum by Pinton et al. (2009) showed that 5 week old piglets fed 2.85 mg DON/kg feed had a 40% decrease of Claudin-4 expression (which was more pronounced in the villi) in samples from DON exposed animals when compared with controls animals [18]. Together, these studies suggest that DON exposure impacts expression of select TJ proteins and barrier function. Our research showed that piglets fed 3.80 ppm DON-contaminated feed starting at weaning for 24 days showed significantly reduced mRNA expression for only CLDN-7 in the ileum (but not for CLDN-1, -2, -3, -4, -6, -10, -23 or OCLN or ZO1) compared to piglets fed the control diet. However, the surface localization of CLDN-1, -3, -4 and -7 (as analyzed by immunohistofluorescence) did not show a difference in the villi or the crypts of jejunal or ileal tissues, regardless of the diet. Consequently, we speculate that any alteration in the intestinal architecture induced by both low level DON exposure (control diet) and higher-level DON exposure may be largely ameliorated over time. 

The effect of DON on the piglet immune system in the gut is also unknown and variable results are described in the literature. An in vivo study showed that feeding 2.2–2.5 mg/kg DON-contaminated diet to pigs (starting weight approx. 11 kg) for 5 weeks had no notable effect on the mRNA expression of TGF-β, IFN-γ, IL-4 and IL-6 in the ileum [11]. In contrast, Becker et al showed that piglets (11.4 kg) fed 1.2 mg DON/kg for 41 days and then 2 mg DON/kg feed for 42 days responded with down-regulation in the expression of IL-1β, IL-8 and TNFα in the blood and down-regulation of IL-1β and IL-8 in the ileum [12]. This result again conflicts with another study where 4-week old piglets fed 0.9 mg/kg DON for 10 days had increased expression of IL-10 and IL-1β genes in the duodenum but expression was slightly down-regulated in the jejunum compared to piglets fed a control diet [9]. Others showed that 5-week old piglets fed a diet artificially contaminated with DON (3 mg/kg) for 35 days did not significantly modulate animal weight but they did result in significant upregulation of immune response genes IL-1β, IL-2, IL-6, IL-12p40 and MIP-1β in the jejunum and a significant induction of the expression of TNF-α, IL-1β and IL-6 in the ileum revealing the presence of active inflammation in the intestine [10]. Consistent with our results, Lessard et al., 2015 showed that 4-week-old piglets fed control diet or diet contaminated with 3.5 mg DON/kg did not show altered mRNA expression levels of proinflammatory cytokines IL1β, IL10, IL12β, and TNF-α in intestinal tissues [23]. In contrast to our study, they showed that pigs fed DON diet had significant up-regulated IFNγ and IL-8 in the ileum compared to control group [23] whereas our data shows that IL-8 showed a trend towards increased expression in DON-fed piglets in ileum after 7 days relative to the control diet fed piglets (*p* < 0.0535). IL-8 is a pro-inflammatory cytokine, which transmits the danger signals to the underlying local antigen-presenting cells and lymphocytes in the gut tissue. Together, these studies may suggest that the duration of DON exposure, as well as the dose and age of initial exposure, may significantly affect the modulation of genes regulating intestinal immune function.

## 4. Conclusions

This study indicates that feeding weaner piglets a diet with a high level of DON contamination (3.30 to 3.80 ppm) resulted in only modest effects on piglet gut health, immune response and body weight, compared to a diet with 0.20 to 0.40 ppm. With the exception of serum cytokine levels, the majority of the molecular analysis in the present study was performed on tissue collected at the end of a dietary treatment period, and as such the effect of DON on other molecular physiology in the acute period is not known. Our results do however suggest that if such an early effect occurred, subsequent compensatory mechanisms were capable of re-establishing intestinal homeostasis. It may, therefore be necessary to evaluate slow introduction of DON-contaminated feed to allow animal sufficient time to adapt without a negative impact on growth and performance.

## 5. Materials and Methods

### 5.1. Animal Care and Selection

All animals used in these experiments were cared for and monitored according to Prairie Swine Centre, Inc.’s (Saskatoon, SK, Canada) Standard Operating Procedures and the experiment was approved by the University of Saskatchewan Animal Research Ethics Board (Protocol #20130054) for adherence to guidelines outlined by the Canadian Council on Animal Care (2009). Date of approval: 8 March 2016.

A total of 24 newly weaned pigs (Camborough Plus x C3378; PIC Canada Ltd., Winnipeg, MB, Canada) were used for this experiment over 4 blocks (12 pigs/treatment). Piglets were weaned at 21 ± 2 (mean ± SD) days of age and 5.89 ± 0.33 kg body weight from sows consuming a commercial lactation diet (Prairie Swine Centre, Inc, Saskatoon, SK, Canada). Upon weaning pigs were placed on a common commercial starter diet for the first 3 d. The pigs were checked twice daily for any signs of ill health. On d 4 post-weaning, pigs were moved to metabolic crates (1.5 × 1.5 m) with plastic-coated, expanded metal floors, polyvinyl chloride walls (0.9 m high) and Plexiglas windows (0.3 × 0.3 m). Pigs were housed individually and remained in the metabolic crates for the duration of the study. Each pen had a bowl drinker and a single-spaced dry feeder providing *ad libitum* access to water and feed. Lights were on from 07:00 h to 19:00 h. The initial room temperature of 26 °C was decreased to 24 °C after 2 weeks and this temperature was maintained for the following 3 weeks.

### 5.2. Dietary Treatments and Preparation

Pigs were randomly assigned to 1 of the 2 dietary treatments within each block in a randomized complete block design. Diets were wheat and barley-based and were formulated based on a 3-phase feeding program to meet or exceed nutrient requirements according to NRC (2012). Pigs were fed a control diet formulated to contain 0 mg/kg DON or treatment diets formulated to contain 4 mg/kg DON (Table 3). 

Phase I was fed for the first 4 days, phase II for the subsequent 2 weeks and phase III for 4 days. The DON-contaminated diet was produced by replacing clean wheat with an amount of DON-contaminated wheat to achieve a final concentration of 4 mg/kg feed. The DON-contaminated wheat was obtained from a single contaminated field in Saskatchewan, Canada. The DON content of the wheat was concentrated by sorting with a BoMill TriQ (BoMill AB, Vintrie, Sweden) NIR seed sorter which produces a wheat fraction with highly consistent level of DON contamination (Kautzman et al., 2015). DON content was determined using HPLC-tandem MS at Prairie Diagnostic Services (Saskatoon, SK, Canada). The mycotoxin composition of DON wheat used for the study is described in Table 4. Samples of each diet were obtained throughout the feeding trial and a composite sample was analyzed (Central Testing Laboratory in Winnipeg, Winnipeg, MB, Canada) for moisture (AOAC 930.15), dry matter, crude protein (AOAC 990.03), Ca (AOAC 968.08), P (AOAC 968.08), Na (AOAC 968.08), NDF (ANKOM) and DON (ELISA DON-V, Vicam, Nixa, MO, USA. 65714). 

Analyzed DON concentrations were 0.3, 0.2 and 0.4 mg/kg for the control diets for Phase I, II and III, and 3.3, 3.8 and 3.8 for the contaminated diets for Phase I, II and III, respectively. This level of variation among diets is typically observed in trials similar to this and attributed to sampling.

### 5.3. Animal Sampling and Weight Calculations

Body weight and feed intake (adjusted for wastage) were determined on day 0, 4, 7, 14, 21, and 24 of the study for the calculation of ADG and ADFI. Blood samples were obtained via jugular venipuncture on d 3, 7, 14, 21, and 25 for the determination of serum cytokine levels (IFN-γ, TNF-α, IL-1β, Il-6, IL-8, IL-10, IL-13) as a measure of overall immune status. On d 25, pigs were euthanized via non-penetrating captive bolt followed by exsanguination. Tissues were obtained from the small intestine (jejunum and ileum). Jejunum was defined as the mid-point of the small intestine and the ileum was defined as 1 m from the ileo-caecal junction. 

### 5.4. Histology and Immunohistoflourescence

Two samples of gut tissue were obtained per site and stored in 10% neutral buffered formalin or snap frozen in dry ice and stored at −20 °C until further analysis. Tissue sections were fixed in 10% neutral buffered formalin for 36 h prior to processing and paraffin embedding. Samples were sectioned at 0.4 µm and mounted on slides (Superfrost Plus, ThermoFisher Scientific, Burlington, ON, Canada), deparaffinized in xylene and rehydrated to distilled water through decreasing concentrations of ethanol.

For histology, tissues were stained with hematoxylin and eosin following standard procedures. Villous height and width were measured and crypt depth was recorded for representative images (data not shown).

For immunohistofluorescence, heat-induced antigen retrieval was carried out in Tris-EDTA buffer (10 mM Tris, 1 mM EDTA Solution, 0.05% Tween 20, pH 9.0) for 30 min at 90 °C prior to blocking in 5% (*w*/*v*) skim milk in PBS for 3hrs at room temperature. Immunohistofluorescent staining was carried out on two non-concurrent tissue sections from each sample with either 1:100 rabbit αCLDN1 (ab15098), 1:200 rabbit αCLDN3 (ab15102), 1:400 dilution of rabbit αCLDN4 (ab53156) or 1 in 200 rabbit αCLDN7 (ab27487). Primary antibodies were diluted in an incubation buffer consisting of 1% *w*/*v* BSA, 1% *v*/*v* Donkey Serum, 0.5% *v*/*v* triton X-100 in PBS and samples stained over night at 4 °C. Slides were then washed three times in PBS and incubated in a 1:400 dilution of Alexa555-conjugated goat α rabbit IgG (ab150082) in incubation buffer for 4 hrs at room temperature. Slides were again washed before counter staining in 0.5 µg/mL DAPI in methanol for 10 min at room temperature prior to cover slipping with Mowiol. Imaging was carried out on an Axiovert 200 M with a 63X neoFluor objective (Zeiss, Oberkochen, Germany) under oil immersion, with a minimum of 4 representative images captured of both the intestinal villi and crypt. Fluorescent images had their background fluorescence subtracted using ImageJ [24].

### 5.5. Bioplex Cytokine Assays

Bioplex bead coupling was performed as per the manufacturer’s instructions. The reagents are listed in Table 5. The multiplex assay was carried out in a 96 well Grenier Bio-One Fluotrac 200 96F black (VWR, #82050-754), which allows washing and retention of the Luminex beads. The 5 beadsets conjugated with the capture antibodies were vortexed for 30 s followed by sonication for another 30 s to ensure total bead dispersal. Bead density was 1200 beads per μl in PBS-BN (1x PBSA pH 7.4 + 1% BSA (Sigma-Aldrich A7030) + 0.05% sodium azide (Sigma-Aldrich, Oakville, ON, Canada). One μL of each beadset was added to 45 μL of diluent (PBSA + 1% New Zealand Pig Serum (Sigma-Aldrich P3484) + 0.05% sodium azide), which was added to each well. The plate was then washed using the Bio-Plex Pro II Wash Station (BioRad, Mississauga, ON, Canada; wash 2 X 100 μL PBST). The porcine IL1β, porcine IL8, porcine IL13, porcine TNFα and porcine IFNγ protein standards were added to the wells at 50 μL per well at a starting concentration of 5000 pg/mL, 200 pg/mL, 5000 pg/mL, 5000 pg/mL and 5000 pg/mL respectively with 2.5 fold dilutions done to produce the standard curve. Sera were pre-diluted 1:4 in diluent and added to the wells at 50 μL per well. 

The plate was sealed with plate sealer (ThermoFisher Scientific, #12565491) and covered with a foil lid. The plate was agitated at 800 rpm for 1 h at room temperature. After 1 h incubation with serum, the plate was washed (3 × 150 μL PBST). Fifty μl of a biotin cocktail consisting of commercially purchased biotins each at 0.5 μg/mL, in house biotinylated anti IL8 at 1/500 and in house biotinylated anti IFNγ at 1/400 was added to each well. The plate was again sealed, covered and agitated at 800 rpm for 30 min at room temperature then washed again as indicated above. Fifty μL of Streptavidin RPE (ProZyme (Cedarlane) PJRS20, Burlington, ON, Canada); diluted to 5 μg/mL) was added to each well. The plate was again sealed, covered and agitated at 800 rpm for 30 min at room temperature and washed as indicated above. A 100 μL of 1x Tris-EDTA was added to each well and then the plate was vortexed for 5 min before reading on the BioRad BioPlex 2000 instrument following the manufacturer’s instructions as described in (Anderson et al., 2011). The instrument was configured to read beadsets in regions 26, 27, 34, 43, and 52 for IL1β, IL8, TNFα, IFNγ and IL13, respectively. A minimum of 60 events per beadset were read and the median value obtained for each reaction event per beadset. For all samples the multiplex assay MFI data was corrected by subtracting the background levels. The lower limit of detection for each cytokine was 32 pg/mL for IFNγ, 80 pg/mL for IL1β and IL-13, 8 pg/mL for IL-8 and 200 pg/mL for TNFα.

### 5.6. Quantitative Gene Expression Analysis

We reduced the number of animals used in the molecular portion of this experiment to allow a greater number of targets to be assessed with both qPCR and by IHF. Animals used for molecular assessment were selected randomly from each of the 4 experimental batches. Jejunal and Ileal tissues samples were ground with mortar and pestle to a fine powder under liquid nitrogen. Total RNA was then extracted using Trizol (Life Technologies, Carlsbad, CA, USA) as per the manufacturer’s directions. DNA contamination was removed using the TURBO DNA-free kit (Life Technologies) before RNA quantity was determined on a NanoDrop spectrophotometer ND-1000 (NanoDrop, Wilmington, DE, USA). RNA integrity was then evaluated on a 1.2% (*w*/*v*) denaturing agarose gel to verify a clear ribosomal RNA banding pattern. Reverse transcription (RT) was done on 2 µg of total RNA using the High Capacity cDNA Reverse Transcription Kit (Life Technologies) before diluting to a final concentration of 10 ng/µL equivalent cDNA. Quantitative real-time polymerase chain reaction (qPCR) was then carried out, in duplicate, using 20 ng of equivalent cDNA, Kappa SYBR fast mastermix (Kapa Biosystems, Wilmington, MA USA) and a primer concentration of 0.75 μM on a Step-One-Plus real time system (Life Technologies, (ThermoFisher Scientific)). Real time primer sets for each gene of interest were designed against RefSeq data obtained from NCBI (Table 6). Where possible, primers were designed to span exon-exon junctions as identified by BLAST Like Alignment Tool (BLAT) comparison with SusScrofa10.2 genomic build. The PCR efficiency for each primer probe set was evaluated against a serial dilution of pooled samples, and found to be greater than 95% for targets. Finally, the data was normalized to the geometric mean of four stable housekeeping genes (ACTB, B2MI, HPRT and RPL19). Data are presented in the form of fold change (2^−ΔΔCt^) relative to the control group within tissue.

### 5.7. Statistics

Growth performance data was analyzed using the MIXED procedure of the SAS statistical program (SAS 9.4, SAS Institute Inc., Cary, NC, USA). Treatment and block were included as fixed effects, pig was included as a random effect, and data were analyzed as repeated measures. The optimal variance structure was determined using the fit statistics within SAS. Differences were between means were determined using the Tukey test and considered statistically significant at *p* ≤ 0.05. A trend towards significant was considered at *p* < 0.10. Statistical analysis of gene expression and serum cytokine results was carried out with a nonparametric Kruskal-Wallis examining preselected comparison of means of the treatment vs. control within tissue or sera or across time.

## Figures and Tables

**Table 1:** Body weight (kg) of piglets assigned to receive either a low-DON control diet or a DON-contaminated diet for 24 days. Piglets were weaned at 21 days of age (experimental day 0).

Time	Control Diet (0.20 to 0.40 ppm DON)	DON-Contaminated Diet (3.30 to 3.80 ppm DON)
Day 0	5.80	5.79
Day 3	5.85	5.77
Day 7	6.09	5.82
Day 14	7.43	6.94
Day 21	9.96	9.28
Day 24	11.43 *	10.39 *

Data are LSMeans. Analysis by repeated measures, overall effect of treatment *p* = 0.04, SEM 0.144; treatment by day, *p* = 0.003, SEM 0.277. * Day 24, *p* < 0.01.

**Table 2:** Growth and feed intake of piglets assigned to receive either a low-DON control diet or a DON-contaminated diet for 24 days. Piglets were weaned at 21 days of age (experimental day 0).

Interval	Control Diet (0.20 to 0.40 ppm DON)	DON-Contaminated Diet (3.30 to 3.80 ppm DON)
Average daily gain (d/g)		
Day 3–7	63.3	6.4
Day 7–14	190.8	158.3
Day 14–21	358.9	331.6
Day 21–24	367.0	273.6 *
Average daily feed intake (g/d)		
Day 3–7	137.6	90.4
Day 7–14	236.9	202.4
Day 14–21	519.7	457.9
Day 21–24	700.8 *	602.3 *

Data are LSMeans. Analysis by repeated measures, ADG, overall effect of treatment, *p* < 0.001, SEM 14.95, treatment by day, *p* = 0.19, SEM 33.28; ADFI, overall effect of treatment *p* < 0.05, SEM 21.11, treatment by day, *p* = 0.46, SEM 33.14. * Day 21–24, ADG, *p* < 0.05; ADFI, *p* < 0.10.

**Table 3:** Ingredient composition (%, as-fed) and calculated and analyzed nutrient content of experimental diets.

Ingredient	Phase I		Phase II		Phase III	
	Control Diet	DON-Contaminated Diet	Control Diet	DON-Contaminated Diet	Control Diet	DON-Contaminated Diet
Wheat (clean)	58.1	20.3	42.6	4.3	44.4	6.2
Wheat (DON)	-	34.8	-	34.8	-	34.8
Soybean meal	22.0	25.0	21.0	24.6	18.6	22.1
Barley	-	-	27.9	27.9	31.9	31.9
Whey	11.4	11.4	-	-	-	-
Fish meal	3.9	3.9	3.2	3.2	-	-
Canola oil	1.9	1.9	2.4	2.4	2.0	2.0
Limestone	1.05	1.05	1.30	1.30	1.55	1.55
Salt	0.40	0.40	0.40	0.40	0.40	0.40
L-Lys, HCl	0.615	0.568	0.573	0.508	0.637	0.575
DL-Met	0.125	0.180	0.105	0.105	0.050	0.050
L-Thr	0.180	0.125	0.175	0.175	0.130	0.130
L-Trp	0.057	0.057	0.004	0.004	0.021	0.021
Choline chloride	0.08	0.08	0.08	0.08	0.08	0.08
Copper sulfate	0.04	0.04	0.04	0.04	0.04	0.04
Vit/min premix ^1^	0.20	0.20	0.20	0.20	0.20	0.20
Calculated nutrient content						
DM (%)	88.7	88.8	87.6	87.7	87.8	87.9
CP (%)	23.5	23.1	22.1	21.8	19.7	19.4
ME (kcal/kg)	3323	3323	3270	3273	3225	3228
Lys (% SID)	1.50	1.50	1.35	1.35	1.23	1.23
Ca (%)	0.73	0.74	0.72	0.73	0.66	0.67
P (%)	0.58	0.59	0.51	0.52	0.42	0.43
DON (ppm)	0.00	4.00	0.00	4.00	0.00	4.00
Analyzed nutrient content						
DM (%)	89.2	89.2	89.5	89.2	89.1	89.4
CP (%)	22.4	23.4	21.8	22.7	19.6	20.8
Ca (%)	0.80	0.88	0.82	1.00	0.86	0.94
P (%)	0.61	0.61	0.53	0.51	0.45	0.46
DON (ppm)	0.30	3.30	0.20	3.80	0.40	3.80

DM, dry matter; ME, metabolizable energy; CP, crude protein. ^1^ Provided per kg of complete diet: Vitamin A, 12,000 IU/kg; Vitamin D 1500 IU/kg; Vitamin E, 70 IU/kg); menadione, 5 mg/kg; Vitamin B12, 0.04 mg/kg; thiamine, 2 mg/kg; biotin, 0.2 mg/kg; niacin, 40 mg/kg; riboflavin, 8 mg/kg; pantothenate, 24 mg/kg; folic acid, 1 mg/kg; pyridoxine, 10 mg/kg; Fe, 150 mg/kg, Zn, 150 mg/kg; Mg, 40 mg/kg; Cu, 20 mg/kg; Se, 0.3 mg/kg; I, 1 mg/kg.

**Table 4:** Mycotoxin content of DON-contaminated wheat ^1^.

Mycotoxin	Level (ppb) ^2^
Deoxynivalenol	11,470
3-acetyl-deoxynivalenol	763.9
15-acetyl-deoxynivalenol	<25.0
α-zearalenol	<66.0
Diacetoxyscirpenol	<25.0
HT-2 toxin	107
Nivalenol	59.2
Ochratoxin A	<25.0
T-2 toxin	<25.0
β-zearalenol	<66.0
Zeralenone	<25.0
Aflatoxin B1	<25.0

^1^ Analyzed by HPLC/MS (Prairie Diagnostic Services, Inc., Saskatoon, SK, Canada). ^2^ Values of <25.0 and <66.0 indicates mycotoxin was below limit of detection.

**Table 5:** Bio-Plex cytokine information for detection of pig proinflammatory cytokines in sera.

Cytokine	Capture Antibody; Supplier	Detection Antibody; Supplier; Dilution	Standard; Supplier; Initial Concentration	Bead; Supplier
IL1β	MAb anti porc IL1β/IF2; R & D MAB6811	Goat anti porc IL1β/IF2 biotin; R & D BAF681; 0.5 μg/mL	recombinant porc IL1β/IF2; R & D 681-PI-10; 5000 pg/mL	Region 26; BioRad MC10026-01
IL8	MAb anti sheep IL8 (86.9% homology); AbD Serotec MCA1660	MAb anti porc CXCL8/IL8; R & D MAB5351; biotinylated in house; 1/400 dilution	Recombinant porc IL-8; Kingfisher RP0109S-005; 200 pg/mL	Region 27; BioRad MC10027-01
IL13	Goat anti swine IL-13; Kingfisher PB0094S-100	Goat anti swine IL-13 biotin; Kingfisher PBB0096S-050; 0.5 μg/mL	Recombinant swine IL-13; Kingfisher RP0007S-005; 5000 pg/mL	Region 52 ; BioRad MC10052-01
TNFα	MAb anti porcine TNFα; R&D MAB6902	Goat anti porcine TNF α biotin; R & D BAF690; 0.5 μg/mL	Recombinant porcine TNFα; R & D 690-PT-025; 5000 pg/mL	Region 34; BioRad MC10034-01
IFNγ	MAb anti-porcine IFNγ; Fisher ENMP700	MAb anti-porc IFNγ; Fisher ENPP700; biotinylated in-house; 1/400 dilution	Recombinant porcine IFNγ; Ceiba Geigy (gift); 2000 pg/mL	Region 43; BioRad MC10043-01

**Table 6:** Target, source and primer-specific information for qPCR analysis in piglet gut tissue.

Target	Source	Forward Primer	Reverse Primer	Amplicon Length (bp)	Annealing Temp (°C)
Actin B	Nygard et al., 2007	5′-CACGCCATCCTGCGTCTGGA-3′	5′-AGCACCGTGTTGGCGTAGAG-3′	100	63
ALOX5	XM_001927671.3	5′-TGGCTTCCCCTTGAGTATTG-3′	5′-CAGGTTCTCCATCGCTTTTG-3′	118	62
ALOX5AP	NM_001164001.1	5′-TGGAGCACGAAAGCAAGAC-3′	5′-CACAGTTCTGGTTGGCAGTG-3′	93	60
B2MI	Nygard et al., 2007	5′-CAAGATAGTTAAGTGGGATCG-AGAC-3′	5′-TGGTAACATCAATACGATTT-CTGA-3′	161	58
CLDN1	NM_001244539.1	5′-TCCTTGCTGAATCTGAACACC-3′	5′-ACACTTCATGCCAACAGTGG-3′	108	60
CLDN2	NM_001161638.1	5′-CGTTGCGTGGAATCTTCAT-3′	5′-GGGAGAACAGGGAGGAAATG-3′	119	60
CLDN3	NM_001160075.1	5′-GCCAAAGCCAAGATCCTCTAC-3′	5′-AGCATCTGGGTGGACTGGT-3′	190	60
CLDN4	NM_001161637.1	5′-CAACTGCGTGGATGATGAGA-3′	5′-CCAGGGGATTGTAGAAGTCG-3′	140	62
CLDN6	NM_001161645.1	5′-CTTCATCGGCAACAGCATC-3′	5′-CAGCAGCGAGTCATACACCT-3′	112	60
CLDN7	NM_001160076.1	5′-ATCGTGGCAGGTCTTTGTG-3′	5′-CTCACTCCCAGGACAAGAGC-3′	192	60
CLDN8	NM_001161646.1	5′-GGAGTGCTCTTCGTCCTCAC-3′	5′-CTGCCGTCCAGCCTATGTA-3′	148	62
CLDN10	NM_001243444.1	5′-GCCCTGTTTGGAATGAAATG-3′	5′-AGCACAGCCCTGACAGTATG-3′	103	62
CLDN14	NM_001161642.1	5′-ACGCCTACAAGGACAATCG-3′	5′-AATGAACTCGGTGTGGGAAC-3′	168	62
CLDN23	NM_001159778.1	5′-TGTCTGGCTGAAGGACTCG-3′	5′-CCACAGGAAAGGAAGGTCAC-3′	112	60
IL1b	NM_001005149	5′-AGAAGAGCCCATCGTCCTTG-3′	5′-GAGAGCCTTCAGCTCATGTG-3′	139	62
IL6	NM_214399	5′-ATCAGGAGACCTGCTTGATG-3′	5′-TGGTGGCTTTGTCTGGATTC-3′	177	60
IL8	NM_213867	5′-TCCTGCTTTCTGCAGCTCTC-3′	5′-GGGTGGAAAGGTGTGGAATG-3′	100	62
IL10	NM_214041	5′-GGTTGCCAAGCCTTGTCAG-3′	5′-AGGCACTCTTCACCTCCTC-3′	202	60
LTA4H	NM_001185132.1	5′-CTGGGAAGGAACACCCCTAT-3′	5′-GGGACAGACACCTCTGCACT-3′	118	60
LTC4S	XM_003123645.4	5′-CTACCGAGCCCAAGTAAACTG-3′	5′-GCGTGCGTACAGGTAGATGA-3′	124	60
OCCLN	NM_001163647.2	5′-GAGTACATGGCTGCTGCTGA-3′	5′-TTTGCTCTTCAACTGCTTGC-3′	102	62
TLR2	NM_213761	5′-ACGGACTGTGGTGCATGAAG-3′	5′-GGACACGAAAGCGTCATAGC-3′	101	62
TLR4	NM_001113039	5′-TGTGCGTGTGAACACCAGAC-3′	5′-AGGTGGCGTTCCTGAAACTC-3′	136	60
TNFa	NM_214022	5′-CCAATGGCAGAGTGGGTATG-3′	5′-TGAAGAGGACCTGGGAGTAG-3′	116	60
ZO1	XM_003353439.2	5′-ACGGCGAAGGTAATTCAGTG-3′	5′-CTTCTCGGTTTGGTGGTCTG-3′	111	62
GAPDH	AF017079	5′-CTTCACGACCATGGAGAAGG-3′	5′-CCAAGCAGTTGGTGGTACAG-3′	170	63
HPRT	Nygard et al., 2007	5′-GGACTTGAATCATGTTTGTG-3′	5′-CAGATGTTTCCAAACTCAAC-3′	91	60
RPL19	AF_435591	5′-AACTCCCGTCAGCAGATCC-3′	5′-AGTACCCTTCCGCTTACCG-3′	147	60
SDHA	Nygard et al., 2007	5′-CTACAAGGGGCAGGTTCTGA-3′	5′-AAGACAACGAGGTCCAGGAG-3′	141	58

## Supplementary Materials

The following are available online at www.mdpi.com/2072-6651/10/1/40/s1, Figure S1: Claudin-1 surface localization in piglet ileal villi and crypts in DON-fed and control fed piglets, Figure S2: Claudin-3 surface localization in piglet ileal villi and crypts in DON-fed and control fed piglets, Figure S3: Claudin-4 surface localization in piglet ileal villi and crypts in DON-fed and control fed piglets, Figure S4: Claudin-7 surface localization in piglet ileal villi and crypts in DON-fed and control fed piglets, Figure S5: Claudin-1 surface localization in piglet jejunal villi and crypts in DON-fed and control fed piglets, Figure S6: Claudin-3 surface localization in piglet jejunal villi and crypts in DON-fed and control fed piglets, Figure S7: Claudin-4 surface localization in piglet jejunal villi and crypts in DON-fed and control fed piglets, Figure S8: Claudin-7 surface localization in piglet jejunal villi and crypts in DON-fed and control fed piglets.
