# Supplementary material for: Molecular and Physiological Effects on the Small Intestine of Weaner Pigs Following Feeding with Deoxynivalenol-Contaminated Feed
Source: Toxins (Basel). 2018 Jan 12;10(1):40. doi: 10.3390/toxins10010040 (PMC5793127; doi:10.3390/toxins10010040)
Supplement: Supplementary file 1 [file toxins-10-00040-s001.pdf]

Supplementary Materials: Molecular and Physiological Effects on the Small Intestine of Weaner Pigs Following Feeding with Deoxynivalenol-Contaminated Feed

J. Alex Pasternak, Vaishnavi Iyer Aka Aiyer, Glenn Hamonic, A. Denise Beaulieu, Daniel A. Columbus and Heather L. Wilson

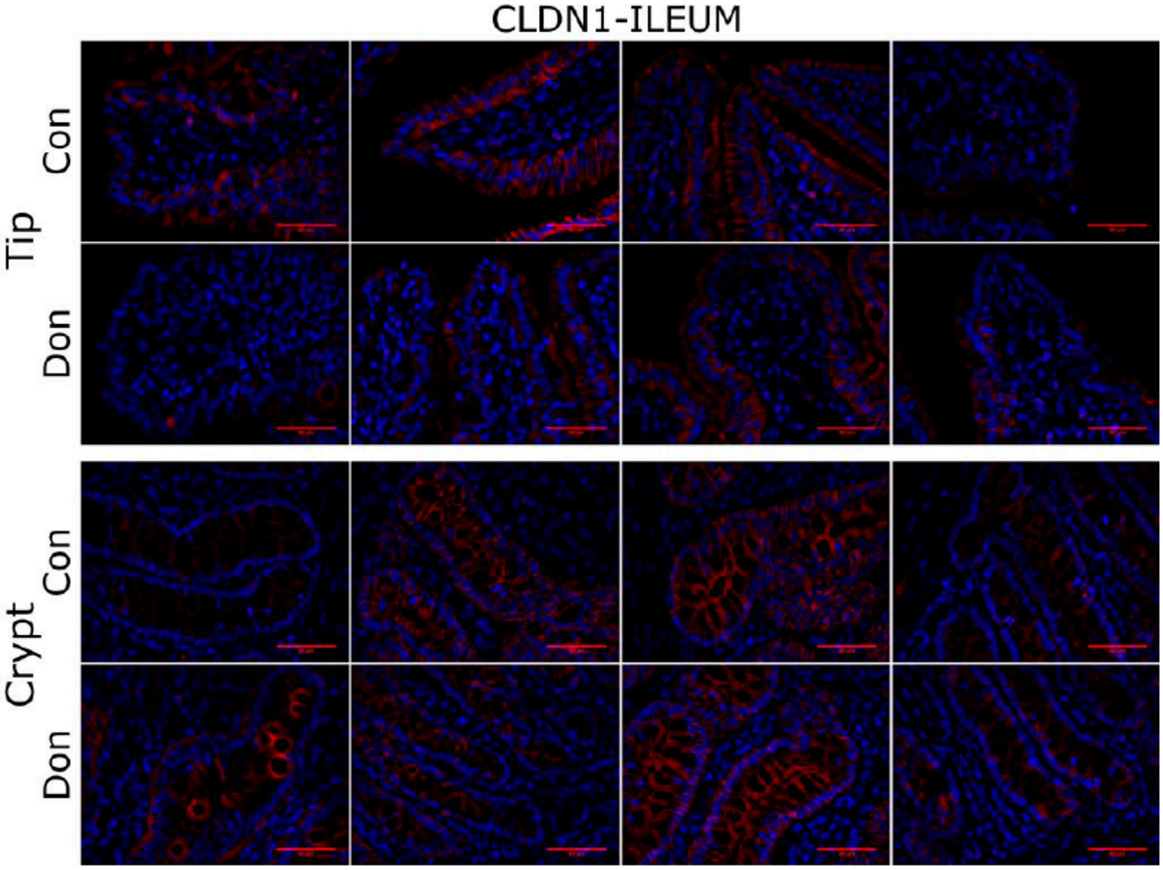

Figure S1. Claudin-1 surface localization in piglet ileal villi and crypts in DON-fed and control fed piglets. Ileal tissue was obtained 25 days after chronic DON-exposure to half of the piglets. CLDN1 was localized to the full length of the pericellular junction within the crypts where as it was found more heavily localized to the apical aspect of the pericellular junction at the villus tip. Secondary antibody: Alexa555-conjugated goat α rabbit IgG (red) in incubation buffer for 4 hrs at room temperature. Nuclear stain: DAPI (blue). Scale bar represents 50 μm.

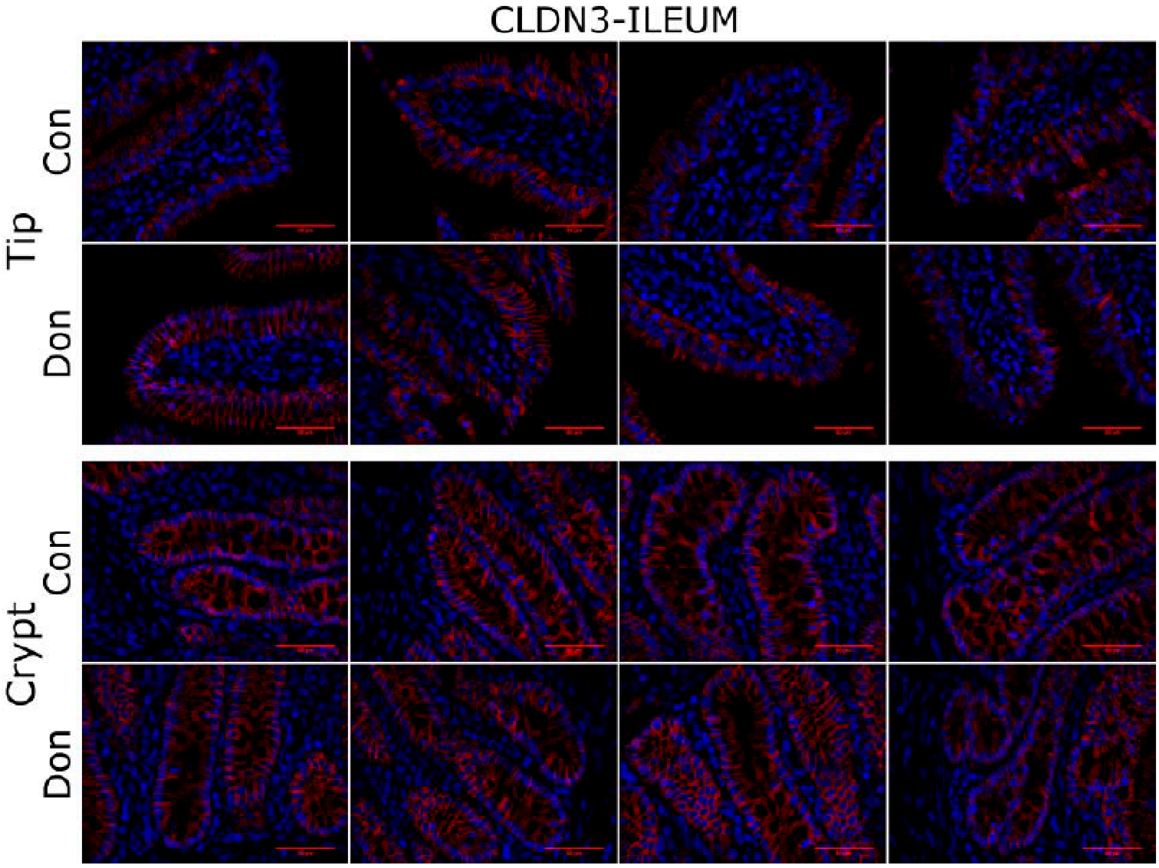

Figure S2. Claudin-3 surface localization in piglet ileal villi and crypts in DON-fed and control fed piglets. **I**leal tissue was obtained 25 days after chronic DON-exposure to half of the piglets. CLDN3 stained the length of the pericellular junction at the villus tip and within the crypts but was more abundant in the latter. Secondary antibody: Alexa555-conjugated goat α rabbit IgG (red) in incubation buffer for 4 hrs at room temperature. Nuclear stain: DAPI (blue). Scale bar represents 50 µm.

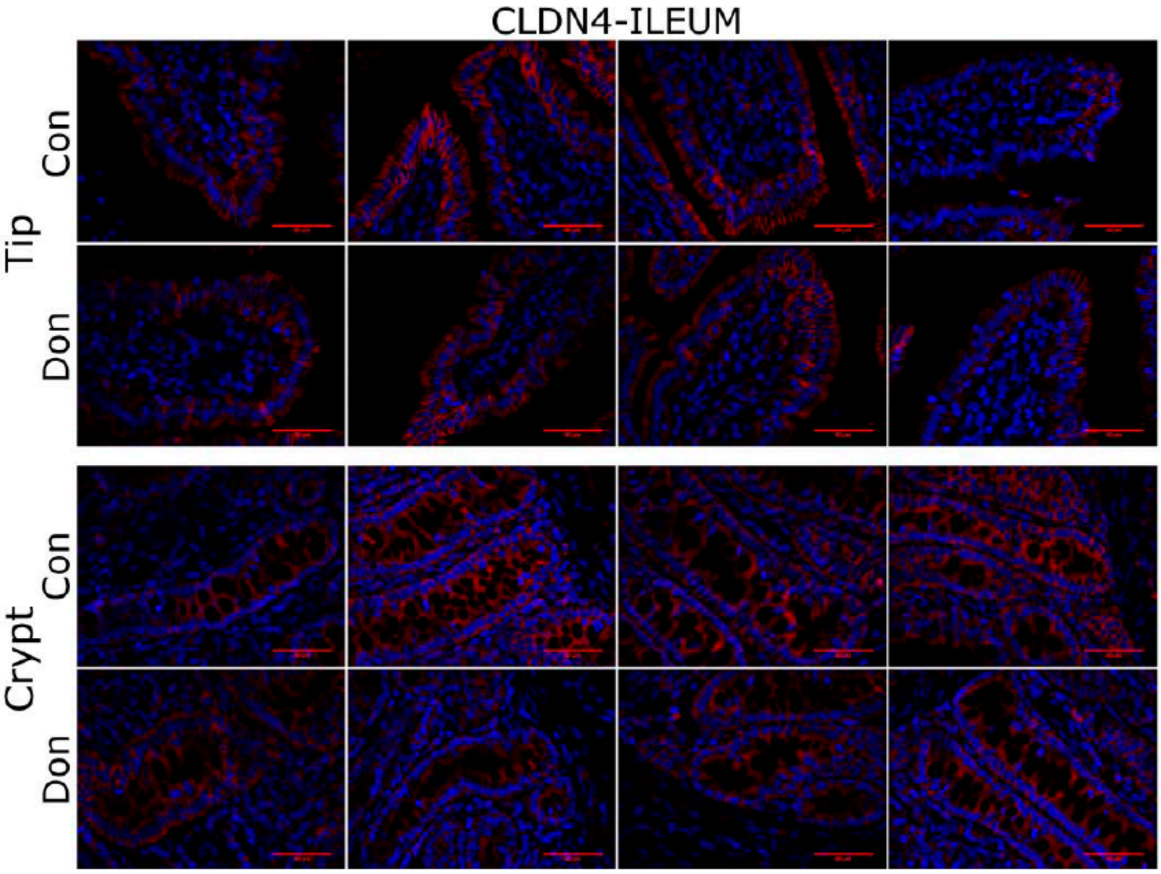

Figure S3. Claudin-4 surface localization in piglet ileal villi and crypts in DON-fed and control fed piglets. **I**leal tissue was obtained 25 days after chronic DON-exposure to half of the piglets. CLDN4 stained the villous surface but was found intracellularly localized in the epithelium of the crypts (C,G,K,O). CLDN7 stained along the length of the pericellular junction at both the villus tip and within the crypts (D,H,LP). Secondary antibody: Alexa555-conjugated goat α rabbit IgG (red) in incubation buffer for 4 hrs at room temperature. Nuclear stain: DAPI (blue). Scale bar represents 50 μm.

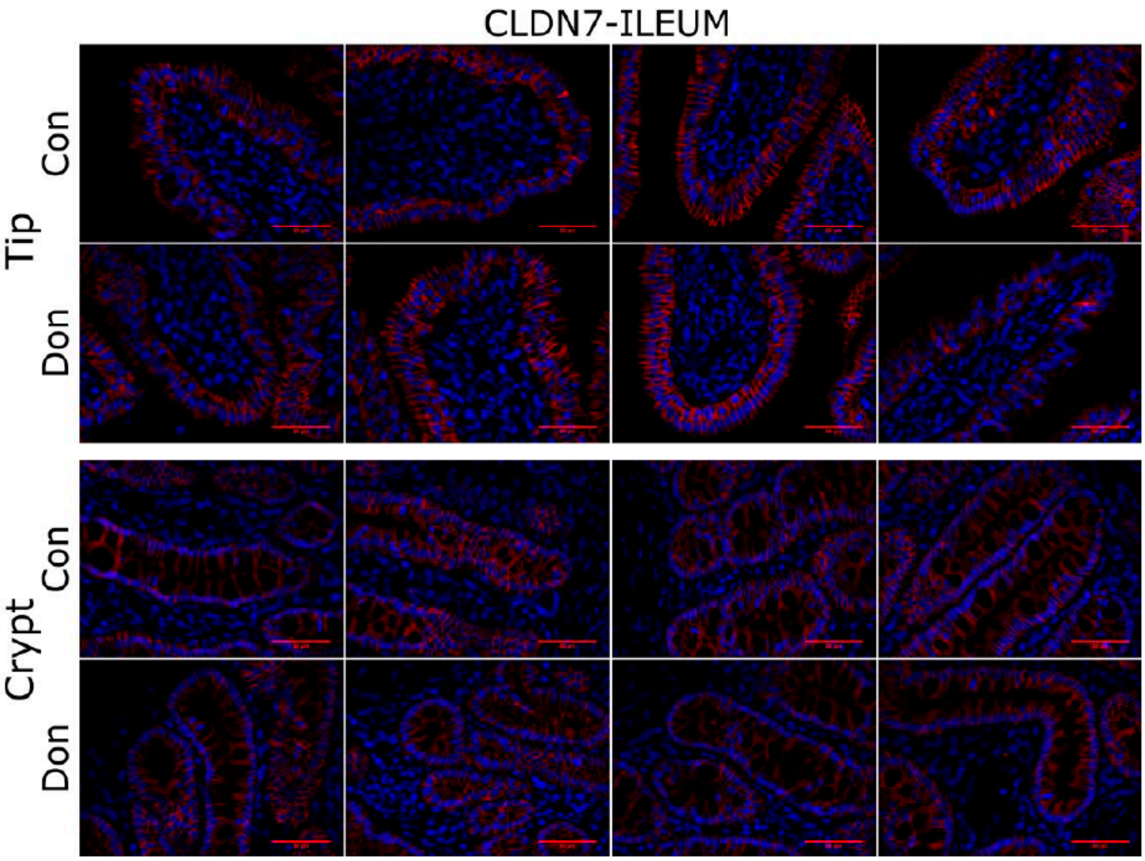

Figure S4. Claudin-7 surface localization in piglet ileal villi and crypts in DON-fed and control fed piglets. Ileal tissue was obtained 25 days after chronic DON-exposure to half of the piglets. CLDN7 stained along the length of the pericellular junction at both the villus tip and within the crypts. Secondary antibody: Alexa555-conjugated goat α rabbit IgG (red) in incubation buffer for 4 hrs at room temperature. Nuclear stain: DAPI (blue). Scale bar represents 50 μm.

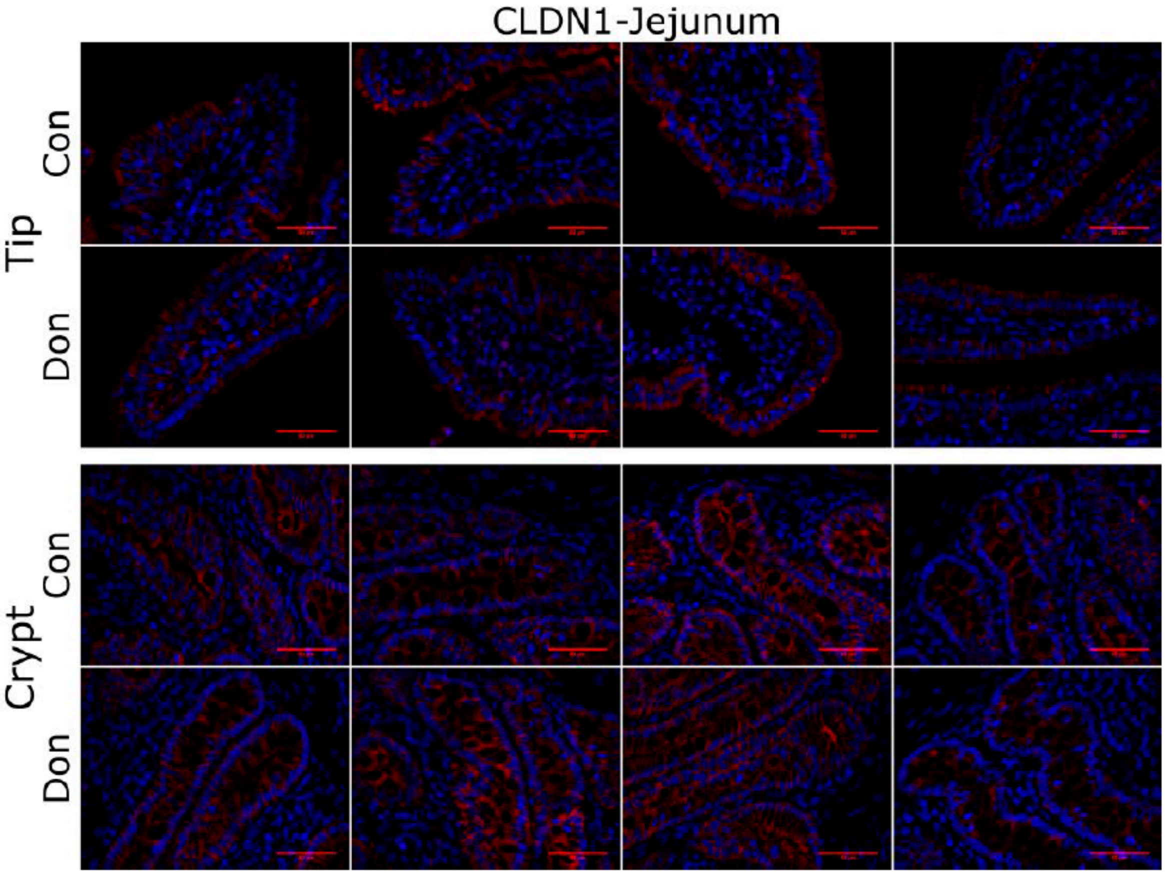

Figure S5. Claudin-1 surface localization in piglet jejunal villi and crypts in DON-fed and control fed piglets. Jejunal tissue was obtained 25 days after chronic DON-exposure to half of the piglets. CLDN1 was localized to the full length of the pericellular junction within the crypts where as it was found more heavily localized to the apical aspect of the pericellular junction at the villus tip. Secondary antibody: Alexa555-conjugated goat α rabbit IgG (red) in incubation buffer for 4 hrs at room temperature. Nuclear stain: DAPI (blue). Scale bar represents 50 μm.

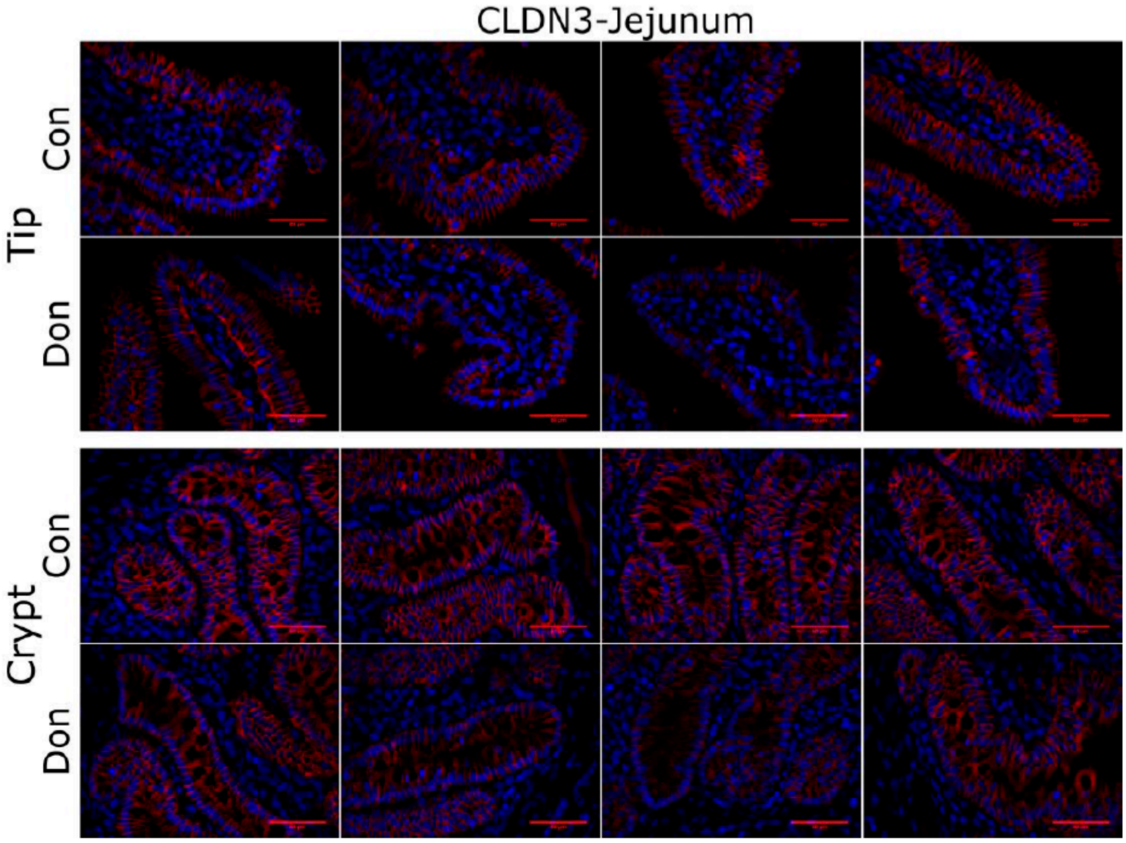

Figure S6. Claudin-3 surface localization in piglet jejunal villi and crypts in DON-fed and control fed piglets. Jejunal tissue was obtained 25 days after chronic DON-exposure to half of the piglets. CLDN3 stained the length of the pericellular junction at the villus tip and within the crypts but was more abundant in the latter. Secondary antibody: Alexa555-conjugated goat α rabbit IgG (red) in incubation buffer for 4 hrs at room temperature. Nuclear stain: DAPI (blue). Scale bar represents 50 µm.

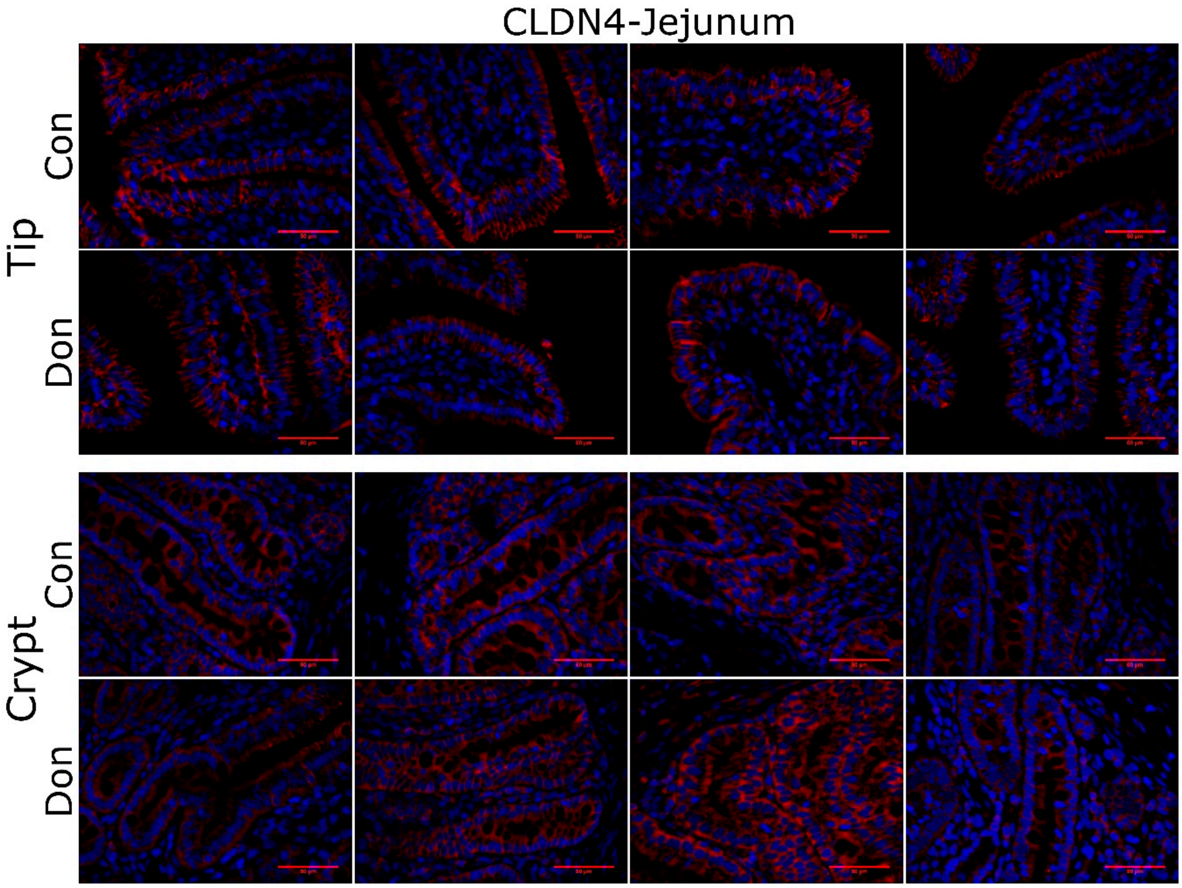

Figure S7. Claudin-4 surface localization in piglet jejunal villi and crypts in DON-fed and control fed piglets. Jejunal tissue was obtained 25 days after chronic DON-exposure to half of the piglets. CLDN4 stained along the length of the pericellular junction at both the villus tip and within the crypts. Secondary antibody: Alexa555-conjugated goat α rabbit IgG (red) in incubation buffer for 4 hrs at room temperature. Nuclear stain: DAPI (blue). Scale bar represents 50 µm.

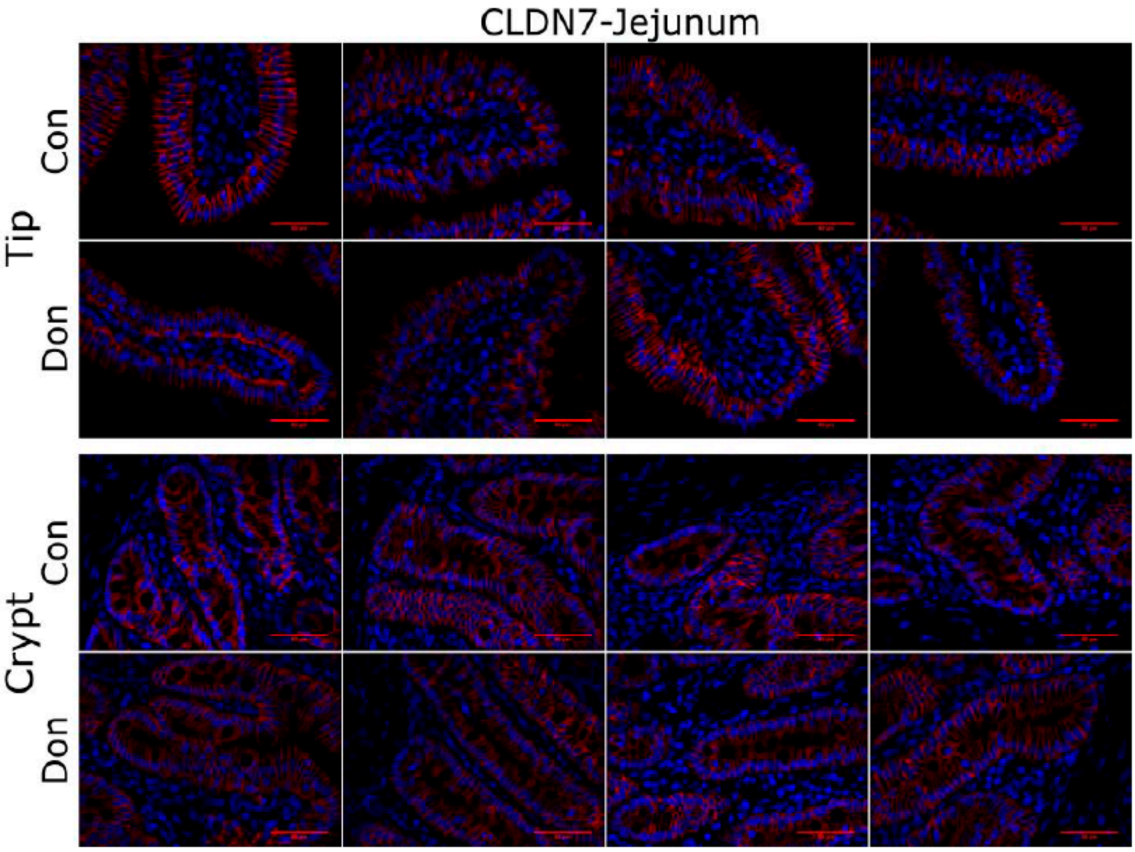

Figure S8. Claudin-7 surface localization in piglet jejunal villi and crypts in DON-fed and control fed piglets. Jejunal tissue was obtained 25 days after chronic DON-exposure to half of the piglets. CLDN7 stained along the length of the pericellular junction at both the villus tip and within the crypts. Secondary antibody: Alexa555-conjugated goat α rabbit IgG (red) in incubation buffer for 4 hrs at room temperature. Nuclear stain: DAPI (blue). Scale bar represents 50 μm.
